# Engineering *Brassica* Crops to Optimize Delivery of Bioactive Products Postcooking

**DOI:** 10.1021/acssynbio.3c00676

**Published:** 2024-02-27

**Authors:** Collin R. Barnum, Myeong-Je Cho, Kasey Markel, Patrick M. Shih

**Affiliations:** † Department of Plant and Microbial Biology, University of California, Berkeley, Berkeley, California 94270, United States; ‡ Department of Plant Biology, University of California Davis, Davis, California 95616, United States; § Biochemistry, Molecular, Cellular and Developmental Biology Graduate Group, University of California Davis, Davis, California 95616, United States; ∥ Innovative Genomics Institute, University of California, Berkeley, Berkeley, California 94720, United States; ⊥ Environmental Genomics and Systems Biology Division, Lawrence Berkeley National Laboratory, Berkeley, California 94710, United States; # Feedstocks Division, Joint BioEnergy Institute, Emeryville, California 94608, United States

**Keywords:** myrosinase, enzyme thermostability, plant synthetic biology, glucosinolate

## Abstract

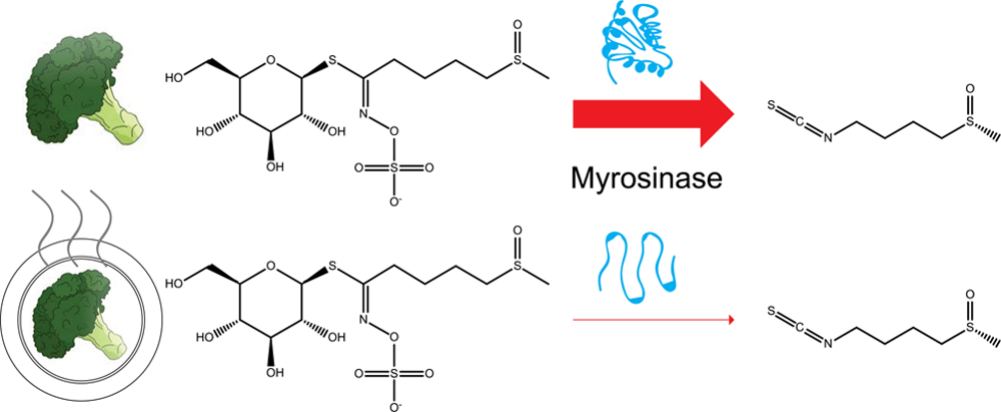

Glucosinolates are plant-specialized metabolites that can be hydrolyzed by glycosyl hydrolases, called myrosinases, creating a variety of hydrolysis products that benefit human health. While cruciferous vegetables are a rich source of glucosinolates, they are often cooked before consumption, limiting the conversion of glucosinolates to hydrolysis products due to the denaturation of myrosinases. Here we screen a panel of glycosyl hydrolases for high thermostability and engineer the *Brassica* crop, broccoli (*Brassica oleracea* L.), for the improved conversion of glucosinolates to chemopreventive hydrolysis products. Our transgenic broccoli lines enabled glucosinolate hydrolysis to occur at higher cooking temperatures, 20 °C higher than in wild-type broccoli. The process of cooking fundamentally transforms the bioavailability of many health-relevant bioactive compounds in our diet. Our findings demonstrate the promise of leveraging genetic engineering to tailor crops with novel traits that cannot be achieved through conventional breeding and improve the nutritional properties of the plants we consume.

## Introduction

Consumption of cruciferous vegetables, such as broccoli, Brussels sprouts, and kale, has been long associated with a reduced incidence of various cancers due to the accumulation of specific small molecules.^1−4^ Specifically, cruciferous vegetables harbor a class of defensive, specialized metabolites derived from amino acids, known as glucosinolates. Glucosinolates are classified into one of three categories, indole, aliphatic, or benzyl, based on the amino acid they are derived from. While glucosinolates are relatively inert, they can be activated through hydrolysis by a class of β-thioglucoside glucohydrolases, called myrosinases, upon tissue disruption to create a variety of bioactive hydrolysis products. The myrosinase-mediated hydrolysis of glucosinolates results in the formation of a variety of hydrolysis products, such as isothiocyanates, nitriles, thiocyanates, epithionitriles, oxazolidin-2-thiones, and epithioalkanes.^5,6^

Due to their variety of structures and reactivity, glucosinolate hydrolysis products have diverse bioactive properties, which are currently under investigation. While the hydrolysis products of all three classes of glucosinolates have potential roles in chemoprevention, the role of isothiocyanates derived from aliphatic glucosinolates has been the most intensely studied. For example, sulforaphane, which is derived from glucoraphanin, has been shown to induce expression of phase II detoxification enzymes, inhibit phase I enzymes, promote cell cycle arrest, and promote apoptosis of cancerous cells;^7^ however, many of these health benefits are still awaiting clinical validation.^8^ Additionally, the hydrolysis products of indole and benzyl glucosinolates have been shown to contribute to the chemopreventive effects of cruciferous vegetables, albeit to a lesser extent than the hydrolysis products of aliphatic glucosinolates.^2^ Due to the reactive nature of these bioactive molecules, they can largely only be obtained from the direct consumption of cruciferous vegetables.

Although glucosinolates are abundant in cruciferous vegetables,^2,3^ the myrosinase-mediated conversion of glucosinolates to bioactive hydrolysis products can be hindered by various food preparation practices, thereby limiting the health benefits of consuming cruciferous vegetables. Factors such as storage, cutting, and cooking can significantly alter the hydrolysis of glucosinolates. In particular, cooking can drastically reduce the conversion of glucosinolates to hydrolysis products due to thermal denaturation of the myrosinase enzyme, reducing the health benefits of consuming cruciferous vegetables. For example, Okunade et al. found that boiling broccoli using a sous vide for 2, 6, and 8 min reduced myrosinase activity by 40%, 90%, and 100%, respectively, displaying the heat sensitivity of broccoli myrosinase.^9^ Additional studies examining the thermal stability of broccoli myrosinase have found that thermal treatment at 70 °C or higher drastically reduces sulforaphane formation.^10,11^ Furthermore, adding exogenous myrosinase in the form of mustard or moringa powder to cooked broccoli leads to sulforaphane formation, providing additional evidence that myrosinase denaturation is responsible for reducing sulforaphane formation.^9,12^ Therefore, identifying strategies to improve the stability of myrosinases to better withstand cooking temperatures could improve the conversion of glucosinolates to bioactive isothiocyanates, enhancing the overall nutritional properties of cooked cruciferous plants.

Traditional breeding efforts have not been able to address these challenges, as plants have never needed to evolve myrosinases that remain functional at boiling temperatures. Such breeding efforts have thus been limited to focusing on increasing glucosinolate content in various crops, such as broccoli;^13^ however, the accumulation of these compounds still is not optimized for bioavailability through human culinary preparation and dietary consumption. Therefore, we sought to enhance the thermal stability of myrosinases in broccoli to enable the conversion of glucosinolates to hydrolysis products following cooking. We screened a panel of bacterial and plant glycosyl hydrolases for thermal stability *in vitro* and identified a myrosinase, TGG4 from *Arabidopsis thaliana*, that displays higher thermal stability than native broccoli myrosinases, making it more suitable for improving glucosinolate hydrolysis following cooking. We then generated transgenic lines of broccoli expressing *TGG4* and demonstrated improved glucosinolate hydrolysis in broccoli following cooking at various temperatures and mastication to simulate standard cooking preparations and ingestion.

## Results and Discussion

### Glucosinolate Hydrolysis Is Limited by Cooking in Wild-Type Broccoli

**Figure 1:**
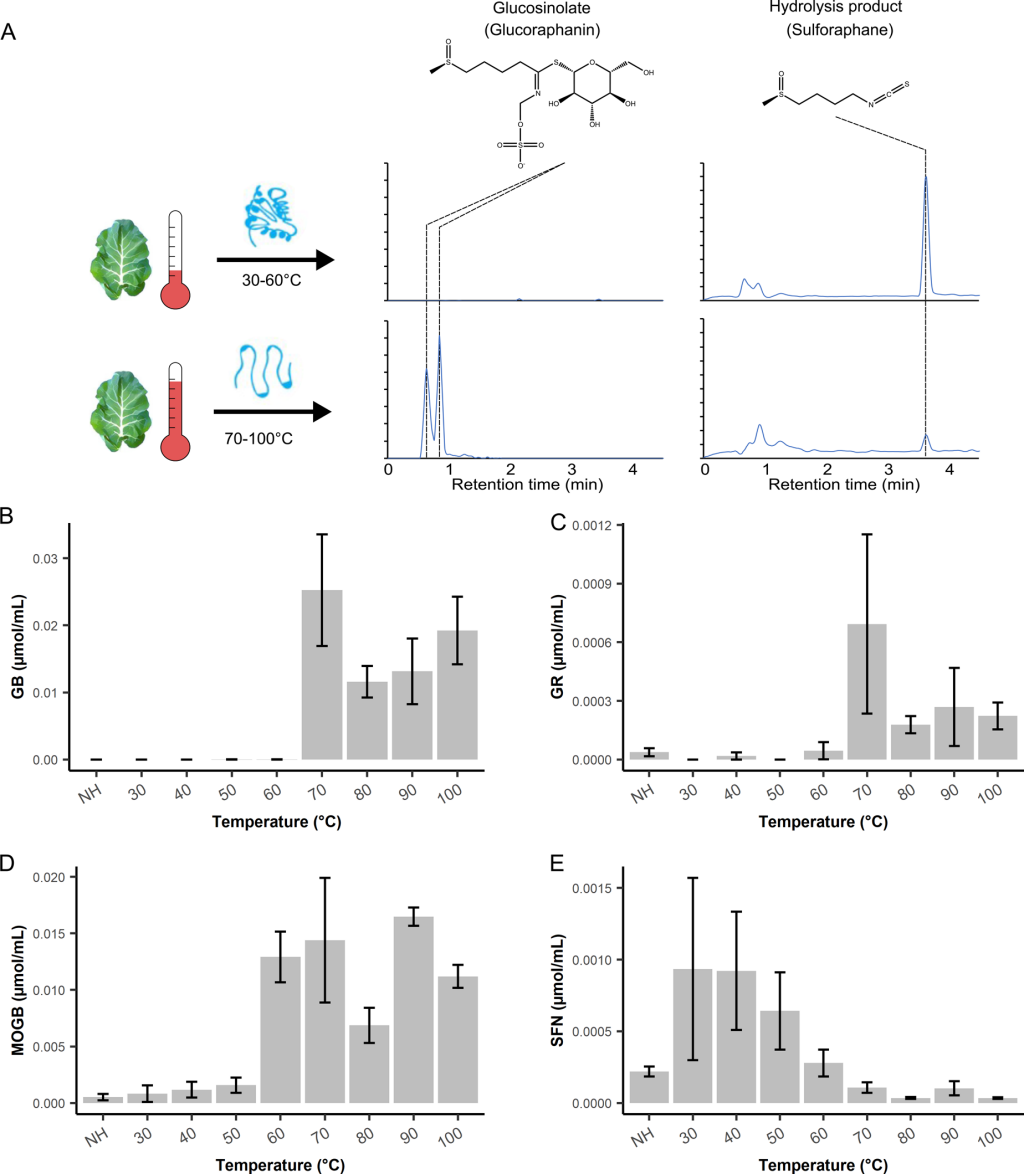
**Myrosinases in *Brassica oleracea* DH1012 are not thermostable.** (A) Diagram illustrating the effects of cooking on the abundance of glucosinolates and hydrolysis products. (B–E) Concentrations of (B) glucobrassicin (GB), (C) glucoraphanin (GR), (D) 4-methoxyglucobrassicin (MOGB), and (E) sulforaphane (SFN) in broccoli leaves treated at various temperatures for 10 min. NH denotes an uncooked control. All bars represent the average value of three leaf cuttings. Error bars represent the standard error of the mean.

To assess the baseline thermal stability of myrosinases in *Brassica oleracea* DH1012, we conducted simulated cooking of broccoli leaves via the sous vide method at various temperatures. *B. oleracea* DH1012 is a double haploid variety of broccoli generated by crossing *B. oleracea* var. *alboglabra* (A12DHd) with *B. oleracea* var. *italica* (Green Duke GDDH33) that was used due to its amenability to *Agrobacterium*-mediated transformation.^14^ However, using this cultivar required the use of broccoli leaves in assays, as DH1012 produces a low amount of floret material. Sous vide in vacuum-sealed bags was chosen as a cooking method to prevent leaching of glucosinolates into the cooking water, as has been previously reported.^15,16^ A cooking time of 10 min was chosen, as a previous study found that cooking broccoli florets between 8 and 12 min at high temperatures was sufficient to inhibit glucosinolate hydrolysis.^17^ Since myrosinases and glucosinolates are compartmentalized in different cell types or cellular compartments,^18,19^ we performed a mastication assay on cooked broccoli to homogenize the broccoli tissue and permit the myrosinase-mediated hydrolysis of glucosinolates, simulating the chewing process (Figure 1A).

The glucosinolate content of broccoli leaves cooked at various temperatures was measured by using liquid chromatography–mass spectrometry (LC-MS). Two indole glucosinolates, glucobrassicin (GB) and 4-methoxyglucobrassicin (MOGB), and one aliphatic glucosinolate, glucoraphanin (GR), were quantified using external calibration curves. The concentration of benzyl glucosinolates was too low in *B. oleracea* DH1012, making detection and quantification unreliable.

GB was fully hydrolyzed in all temperature treatments below 70 °C, including the unheated control (Figure 1B). Interestingly, the concentration of MOGB showed a gradual increase as the temperature of the heat treatment rose, with a stark increase between 50 and 60 °C, indicating that myrosinases were unable to hydrolyze MOGB at temperatures of 60 °C or higher (Figure 1D). This could be attributed to either degradation of an MOGB-specific myrosinase at a lower temperature or alteration of the substrate specificity of the myrosinase by mild heating, limiting its ability to hydrolyze MOGB. While both are possible, part of indole glucosinolate hydrolysis is often attributed to “atypical” myrosinases, such as PYK10 and PEN2, which could denature at temperatures lower than typical myrosinases.^20^

The GR content was low in broccoli leaves heated at temperatures equal to or below 60 °C; however, heating at 70 °C or higher led to drastic increases in the GR content (Figure 1C). Furthermore, the concentration of the GR-derived isothiocyanate, sulforaphane (SFN), showed an inverse relationship to glucoraphanin, with the highest concentrations being reached at temperatures between 30 and 60 °C (Figure 1E). Interestingly, some SFN was still detected in leaves cooked at temperatures above 70 °C; however, this is likely due to the mild tissue damage that occurs when the tissue is prepared for cooking. Together, these results indicate that myrosinases in the leaves of DH1012 lose the majority of their activity when cooked between 60 and 70 °C, in accordance with previously reported findings for broccoli florets.^17^ The loss of myrosinase activity at these temperatures limits the formation of beneficial glucosinolate hydrolysis products.

### Biochemical Screen for Thermostable Myrosinases

Due to the degradation of native broccoli myrosinases following heat treatment above 60 °C, we sought to identify non-broccoli myrosinases with a higher thermal stability. Myrosinases are a subfamily within the broader protein family of glycosyl hydrolases that exhibit thioglucosidase activity. Substrate promiscuity of glycosyl hydrolases has been well-documented, with several studies previously demonstrating that various glycosyl hydrolases exhibit thioglucosidase activity.^21,22^ Additionally, several microbial glycosyl hydrolases have been characterized for their thermal stability.^23,24^ Thus, we utilized a previously characterized panel of diverse family I glycosyl hydrolases from microbes to potentially identify a microbial glycosyl hydrolase that can hydrolyze glucosinolates after high heat treatments.^24^ We expressed 36 microbial family I glycosyl hydrolases with a range of thermal stabilities in *Escherichia coli* BL21DE3* (Figure S1). Additionally, we cloned two previously known myrosinases (TGG4 and I1) and one proposed myrosinase (MF461331) into a binary vector containing a C-terminal His_6_ tag. Transient expression in *Nicotiana benthamiana* was chosen as an expression platform, as it is capable of producing appreciable amounts of heterologous protein and would enable plant-specific protein glycosylation patterns, which have been proposed to have a role in myrosinase stability.^6,25,26^ Expression of TGG4 from *A. thaliana* and I1 from *Armoracia rusticana* produced soluble myrosinase at yields that were suitable for further analysis (Figure S2). Previous work on *B. oleracea* var. *italica* had identified the expression of a putative myrosinase, MF461331, based on homology and expression profiles;^25^ however, transient expression of this gene did not produce an enzyme capable of hydrolyzing the widely available aliphatic glucosinolate sinigrin when expressed in *N. benthamiana* (Figure S3).

**Figure 2:**
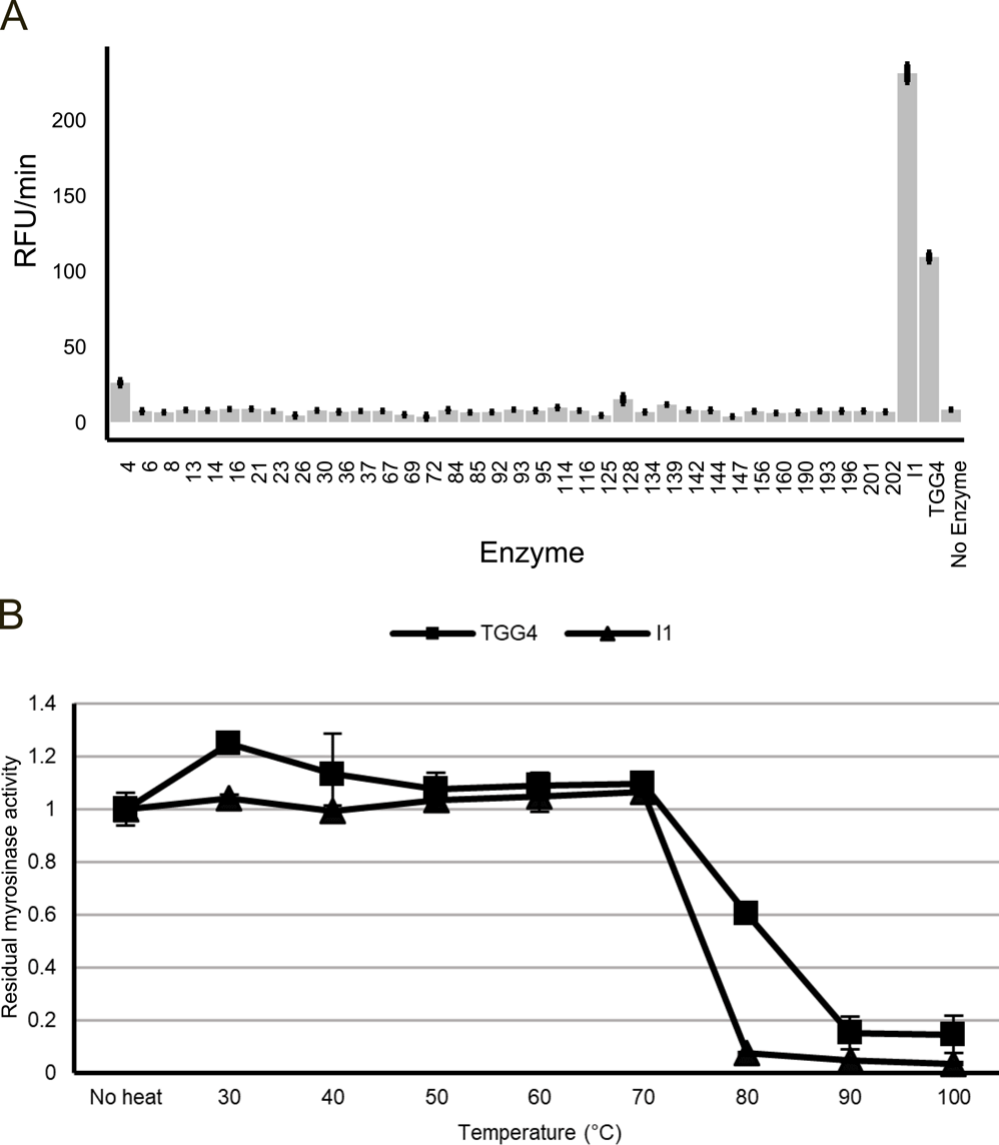
**Biochemical screening and identification of the thermostable myrosinase TGG4.** (A) Screen of 36 purified, thermostable microbial family I glycosyl hydrolases (4–202) and two plant myrosinases (I1 and TGG4) using the Amplex Red Glucose Assay to measure the release of glucose from sinigrin hydrolysis. (B) Residual myrosinase activity of TGG4 and I1 following 5 min heat treatment at various temperatures as determined by the difference in absorbance of light at a wavelength of 230 nm. All bars or dots represent the average value of three technical replicates. Error bars represent the standard error of the mean.

The lysate from the 36 family I glycosyl hydrolases, TGG4, and I1 was purified via immobilized metal affinity chromatography (IMAC) using Ni-NTA agarose beads. Purified enzymes were initially assayed using the Amplex Red Glucose/Glucose Oxidase Assay kit to determine which possessed myrosinase activity by measuring the release of glucose following glucosinolate hydrolysis (Figure 2A). I1 and TGG4 were found to have substantially higher activity than any microbial family I glycosyl hydrolase (Figure 2A), leading us to further assess I1 and TGG4. Purified TGG4 and I1 were tested for myrosinase activity following a 5 min heat treatment. Myrosinase activity was determined by monitoring the myrosinase-mediated degradation of sinigrin via a UV-based plate reader assay, as glucosinolates have been shown to absorb light in the UV range.^27^ Both I1 and TGG4 showed higher thermal stability than native broccoli myrosinases based on glucosinolate degradation data in broccoli (Figures 1B–E and 2). I1 lost activity following a 5 min heat treatment at 80 °C or higher, while TGG4 retained ∼60% of its activity in the 80 °C heat treatment and retained ∼19% of its activity after a 90 or 100 °C heat treatment (Figure 2). While both I1 and TGG4 displayed thermal stability higher than myrosinases present in wild-type broccoli, TGG4 displayed even higher thermal stability than I1. Additionally, TGG4 has been shown to have tolerance for a range of pH and salinity.^28^ Moreover, TGG4 has been demonstrated to accept a diversity of glucosinolate substrates encompassing indole, benzyl, and aliphatic glucosinolates,^28−30^ which are the major classes of glucosinolates in many cruciferous vegetables.^2,31,32^ Thus, TGG4 represents an ideal thermostable myrosinase to engineer into a wide variety of potential *Brassica* crops due to its innate substrate promiscuity and its unique thermostability properties. As such, TGG4 was chosen as a promising candidate for heterologous expression in *B. oleracea* DH1012.

### Generation of Transgenic Broccoli Lines

Due to the loss of native broccoli myrosinase activity in broccoli cooked at temperatures of 70 °C or higher, we sought to improve glucosinolate hydrolysis in cooked broccoli by expressing TGG4 in *B. oleracea* DH1012. Native myrosinase expression is typically limited to specific cell types to prevent unwanted glucosinolate hydrolysis.^18,19^ However, there are limited promoters that have been well-characterized in broccoli to also exhibit the same expression pattern as the endogenous myrosinases, and many typical plant engineering efforts utilize constitutive promoters to ensure the expression of heterologous proteins. To balance these two approaches, we pursued two strategies in parallel: (1) utilize a myrosinase-specific promoter and (2) utilize a constitutive promoter to facilitate overexpression throughout the plant.

**Figure 3:**
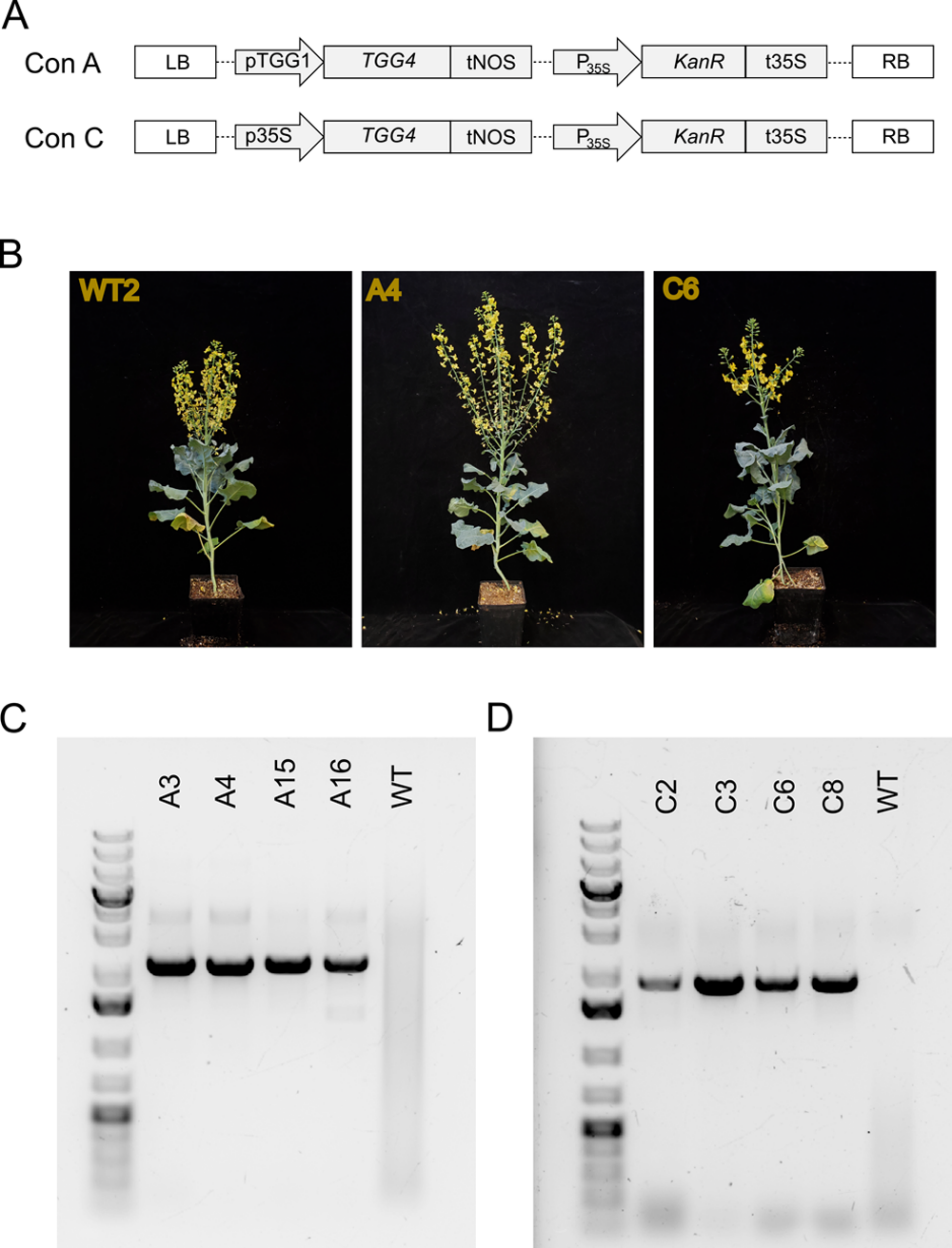
**Generation of transgenic *Brassica oleracea* DH1012.** (A) Constructs used for the creation of transgenic lines. (B) Representative photos of transgenic broccoli. (C, D) Amplification of the promoter–transgene–terminator cassette from transgenic lines. One primer pair was used for the amplification of A line insertions, and a second primer pair was used for the amplification of C line insertions.

pTGG1 is a myrosinase-specific promoter from *A. thaliana* that directs expression to guard cells and phloem idioblasts in *A. thaliana*.^33^ GUS expression driven by pTGG1 in *Nicotiana tabacum* directed expression to the guard cells, suggesting that this promoter may maintain its tissue specificity even in distantly related species.^34^ Since pTGG1 is able to direct expression in a myrosinase-specific manner, we used it to drive expression of TGG4 in an attempt to recreate native myrosinase expression patterns in *B. oleracea* DH1012, generating construct A (Figure 3A). Additionally, to ensure high expression, we generated constructs with TGG4 expression driven by the high-strength constitutive promoter p35S from cauliflower mosaic virus, generating construct C (Figure 3A).

*B. oleracea* DH1012 was transformed with constructs A and C through *Agrobacterium*-mediated transformation as previously described.^35^ Multiple transgenic lines were generated for each construct. These transgenic plants exhibited no notable phenotypes (Figure 3B). Four of these lines from each genotype were subsequently screened for bioassays after transgene insertions were confirmed through PCR and gel electrophoresis (Figure 3C,D). Representative lines were characterized by qPCR (Figure S4).

### Transgenic Broccoli Expressing TGG4 Exhibits Improved Glucosinolate Hydrolysis Following Heat Treatment

To assess the effect of inserting constructs A and C into *B. oleracea* DH1012, we harvested leaves from the wild-type and all generated transgenic A and C lines. We performed simulated cooking at various temperatures, mastication of leaves, and analysis of the leaf glucosinolate content using LC-MS. Transgenic lines transformed with construct A did not display any differences in the glucosinolate content following heat treatment (Figure S5), despite the fact that TGG4 should be able to effectively hydrolyze glucosinolates in treatments up to 80 °C (Figure 2). This could be due to the expression strength of the pTGG1 promoter used in construct A, limiting the amount of TGG4 produced for glucosinolate hydrolysis.

**Figure 4:**
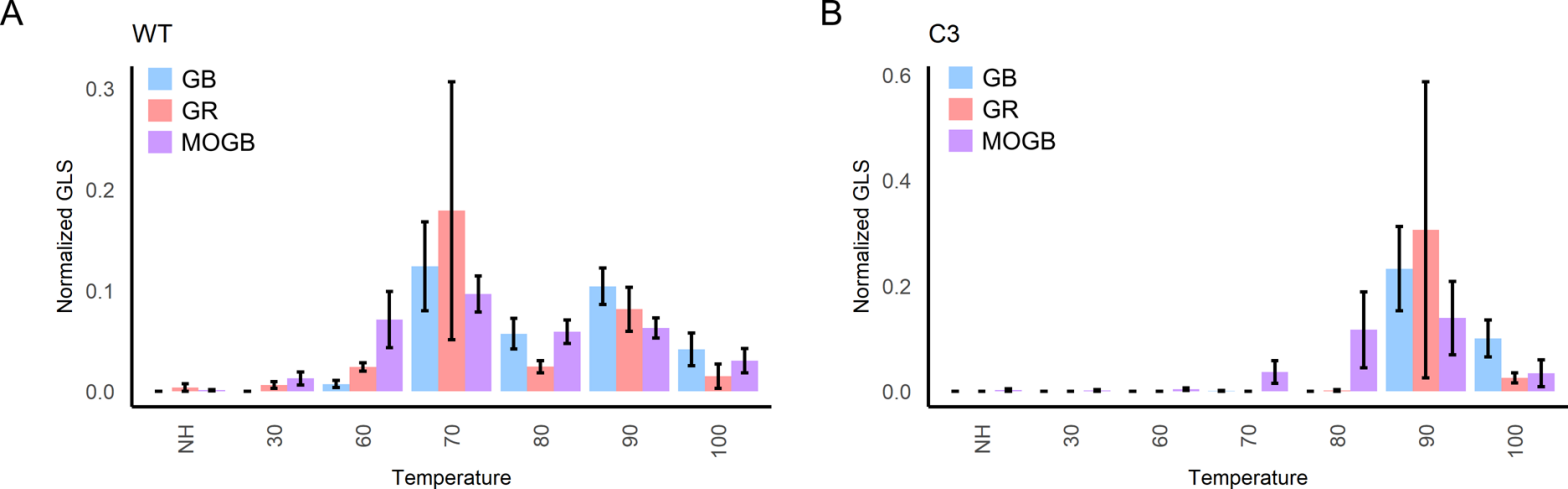
**Heterologous expression of TGG4 in broccoli improves glucosinolate hydrolysis following cooking.** Glucosinolate content of leaves from (A) wild-type plant and (B) transgenic line C3 were analyzed via LC-MS. The glucosinolate content of each line was quantified and normalized to the total amount of a glucosinolate present among all treatments to provide relative concentrations between treatments. GB = glucobrassicin, GR = glucoraphanin, MOGB = 4-methoxyglucobrassicin. All bars represent the average normalized value of three leaf cuttings. Error bars represent the standard error of the mean.

Several transgenic lines transformed with construct C showed substantial changes in the relative glucosinolate content at specific heat treatments compared to wild-type broccoli. Lines C2 and C6 displayed improved glucosinolate hydrolysis for GR and GB at 70 °C compared to the wild type, indicating an improvement in the retention of myrosinase activity following high-temperature heat treatment (Figure S6B,C). Lines C3 (Figures 4B and S6D) and C8 (Figure S6E) displayed reduced normalized concentrations of glucosinolates at 70 and 80 °C for glucoraphanin and glucobrassicin, displaying a 20 °C improvement in the retention of myrosinase activity compared to the wild type. While TGG4 retained some activity after 90 and 100 °C heat treatments *in vitro* (Figure 2), it did not retain activity at 90 or 100 °C in transgenic broccoli (Figure 4). This suggests that TGG4 is capable of effectively managing shorter heat treatments at 90 and 100 °C, similar to the 5 min *in vitro* treatment. However, it appears unable to maintain its activity after a 10 min heat treatment, similar to the case observed in broccoli. Interestingly, all transgenic lines generated with construct C still showed relatively high amounts of MOGB in a manner similar to the wild type. This suggests that TGG4 is unable to hydrolyze MOGB effectively *in planta*, providing further evidence that an endogenous myrosinase may be responsible for the degradation of MOGB.

Overall, these results provide a blueprint of how *Brassica* crops can be engineered to improve the degradation of glucosinolates for the production of beneficial bioactive small molecules. The innate thermal stability of TGG4 enables the production of transgenic broccoli lines that can hydrolyze glucosinolates even when cooked at high temperatures. While TGG4 did not retain activity when sustained at near-boiling temperatures, it is notable that the internal temperature of foods may not immediately or necessarily rise to the temperatures of their external environment. Thus, different cooking methods could produce suitable texture while maintaining a lower core temperature. For example, steaming broccoli for 0.5 to 3 min elevates the core temperature to 45 and 95 °C,^36^ respectively, eliminating the requirement to introduce a myrosinase capable of withstanding 100 °C. Our work provides a transgenic approach to improving the nutritional outcomes of cooked *Brassica* vegetables. More broadly, this work displays the ability to engineer nonmodel crops for improved nutritional quality or postharvest attributes.

## Conclusion

Plants are uniquely intertwined with many facets of human health. Arguably, the most intimate and pervasive way that we interact with plants is through the consumption of complex plant materials, which contain a wealth of bioactive phytochemicals that play a role in shaping our gut microbiota, disease status, and long-term health.^37^ Importantly, the context in which we prepare and consume these valuable phytochemicals plays a critical role in shaping their health properties. Notably, cooking can lead to drastic transformations in the nutritional profiles of foods. While many efforts have focused on breeding and engineering plants to accumulate target compounds, various postharvesting processes (e.g., cooking or fermenting) can lead to the alteration of the chemical profile of foods, often changing the concentration or bioavailability of beneficial compounds. Engineering plants to take these practices into account to optimize the delivery of nutritional compounds through our diet has the potential to improve preventive medicine and overall well-being.

The diversity of bioactive components in plants presents a wealth of opportunities to improve the nutritional quality of plants; however, traditional breeding efforts are unable to engineer plants with completely novel traits. In the case of myrosinase thermal stability, there has been no natural selective pressure to increase the stability of myrosinases to survive cooking temperatures. Transgenic approaches open the door to redefining the scope of the methods to which we can modify plants. In this work, we characterized and expressed an innately thermostable myrosinase in broccoli. This approach increased the temperature at which glucosinolate hydrolysis can occur by 20 °C, thereby improving the conversion of inert glucosinolates to health-relevant bioactive hydrolysis products in cooked broccoli. Future efforts may further optimize this system by utilizing computational tools to design custom enzymes with further improved thermal stability properties to survive longer treatments at boiling temperatures. Overall, this work displays a novel approach to improving the availability of chemopreventive compounds whose formation is typically diminished by cooking.

It has long been proposed that cooking was a pivotal step in human evolution, which enhanced the energy and nutrition available from food. Similarly, early events in crop domestication have become the foundation of our modern agricultural system. As humans continue to evolve with our domesticated crops, our ability to manipulate plants has advanced from conventional breeding to genetic engineering approaches, enabling the introduction of novel traits into crops with increasing precision and ingenuity. Such technological advances may continue our long history and evolutionary trajectory of innovating new ways to manipulate plant genomes for the ultimate purpose of maximizing human health. Given the diversity of both edible plants and culinary practices, there are a wide range of potential applications for leveraging transgenic crops in combination with cooking practices to maximize the nutritional benefits of the foods we consume.

## Methods

### Expression of Glycosyl Hydrolases in *E. coli*

Family I glycosyl hydrolases in pET45B expression vectors were used to transform *E. coli* BL21DE3*. Colonies were picked from freshly transformed plates and used to inoculate 5 mL of terrific broth containing 100 μg/mL ampicillin that was grown overnight at 37 °C. The overnight cultures were then used to inoculate 250 mL of terrific broth containing 100 μg/mL ampicillin in 500 mL baffled flasks. Cultures were shaken at 200 rpm at 37 °C until reaching an OD_600 nm_ of 0.6. Expression was induced by adding isopropylthio-β-galactoside (IPTG) to a final concentration of 1 mM and incubating at 16 °C for 16 h while shaking at 200 rpm. Cells were then pelleted by centrifugation at 5000*g* for 10 min, resuspended in 2.5 mL of 1× phosphate-buffered saline (PBS), and frozen at −80 °C.

### Plasmid Construction and Transient Expression of Candidate Myrosinases

Candidate myrosinases were expressed through transient expression in *N. benthamiana*. Genes for candidate myrosinases were cloned into the binary vector with a C-terminal His_6_ tag, CRB005, using Golden Gate assembly.^38^ Constructs were used to transform XL1-blue *E. coli* competent cells, which were subsequently plated on LB agar plates containing 50 μg/mL kanamycin. Colonies were selected, cultured, and mini-prepped, and the resulting plasmid was sequence-verified. Purified plasmid was then used to transform *Agrobacterium tumefaciens* str. GV3101 via electroporation.^39^ Electroporated cells were plated on LB agar plates with 50 μg/mL kanamycin, 50 μg/mL rifampicin, and 10 μg/mL gentamicin. *A. tumefaciens* str. GV3101 harboring individual candidate myrosinases was grown in LB overnight to an OD_600_ (VWR, V-1200) of 0.8 to 1.2. The cultures were centrifuged at 4000*g* for 10 min, and the supernatant was decanted. Cell pellets were resuspended in infiltration medium (10 mM MES, 10 mM MgCl_2_, 500 μM acetosyringone, pH 5.6) and incubated at room temperature for 1 h with gentle rocking (Thermolyne, VariMix). *A. tumefaciens* strains harboring each glycosyltransferase were mixed in equal amounts alongside a strain harboring the p19 silencing suppressor to reach a final OD_600_ of 0.5. *A. tumefaciens* mixtures were injected into the abaxial side of a leaf on a four-week-old *N. benthamiana* using a needleless syringe.

### Purification of Candidate Myrosinases

Infiltrated *N. benthamiana* leaves were harvested 5 days postinfiltration. A 10 g sample of transiently expressing leaves was ground using a mortar and pestle in liquid nitrogen to a fine powder. The fine powder was then transferred to a 50 mL conical tube and resuspended in 30 mL of protein extraction buffer consisting of 1× PBS, 10 mM imidazole, 10 μg/mL DNase, pH 7.4. Samples were incubated for 1 h at 4 °C on a rocking platform (Thermolyne, Varimix). Following incubation, samples were centrifuged at 10000*g* for 15 min at 4 °C to pellet insoluble plant material. The supernatant was transferred to 35 mL Oak Ridge tubes to be centrifuged at 30000*g* for 10 min at 4 °C. The supernatant was syringe-filtered using a poly(ether sulfone) 0.8/0.2 μm filter (Pall).

Frozen *E. coli* pellets were thawed in 15 mL of lysis buffer while shaking at 30 °C for 10 min. After being fully resuspended, the suspension was sonicated (Fisher Scientific) for 4 min at power level 10 on ice four times in the following pulse pattern: 30 s on, 30 s off. The suspension was transferred to Oak Ridge tubes and centrifuged at 15000*g* for 20 min. The supernatant was then transferred to a 50 mL conical tube for purification.

Candidate myrosinases were purified by using nickel nitriloacetic acid (Ni-NTA) agarose beads. Briefly, 2 mL of Ni-NTA agarose bead slurry was placed in a 20 mL disposable column and washed with 5 mL of elution buffer (1× PBS, 200 mM imidazole, pH 7.4) followed by a 20 mL wash with wash buffer (1× PBS, 10 mM imidazole, pH 7.4). Washed Ni-NTA agarose beads were then mixed with the protein-containing plant extract or *E. coli* extract, placed in a 50 mL conical tube, and incubated with gentle rocking for 1.5 h. Following incubation, the mixture of plant extract and Ni-NTA agarose beads was returned to the 20 mL column and allowed to drain. The Ni-NTA agarose beads were then washed three times with 20 mL of wash buffer. The protein was eluted from the beads using 10 mL of elution buffer, and the flowthrough was collected. Eluted protein was dialyzed twice using dialysis tubing (SnakeSkin, 10000 MWCO, Thermo Scientific) in 1 L of 1× PBS (pH 7.4) at 4 °C for 8 h. Total protein was quantified using a nanodrop (NanoDrop OneC, Thermo) measuring at an absorbance of 280 nm. Protein samples were analyzed by SDS-PAGE using precast 12% polyacrylamide gels (BioRad, mini-PROTEAN TGX).

### Initial Screen of Purified Enzymes for Myrosinase Activity

Purified enzymes were mixed with a 10 mM sinigrin solution resuspended in 1× PBS (pH 7.4) and assayed according to manufacturer’s suggestions using the Amplex Red Glucose/Glucose Oxidase Kit (Thermo Fisher Scientific). The final volume of solution was 50 μL containing 0.1 mg/mL purified enzyme and 5 mM sinigrin, along with the components of the kit at the recommended concentrations. The reaction was monitored every 3 min using a BioTek Synergy H1 plate reader with an excitation wavelength of 530 nm and an emission wavelength of 590 nm in a 384-well plate (Greiner Bio-One UV Star).

### Measuring Myrosinase Activity Following Heat Challenge

Purified myrosinase was assayed for activity by measuring the degradation of glucosinolates over time in 384-well plates suitable for UV-based absorbance assays (Greiner Bio-One UV Star). Each well contained 50 μL of 1× PBS (pH 7.4) containing 0.1 mg/mL purified myrosinase and 1.25 mM sinigrin. Absorbance at 230 nm was measured for each well every 1.5 min using a BioTek Synergy H1 plate reader. Residual activity was calculated by determining the difference in absorbance units at 5 min and normalizing it to the no-heat control.

### Generation of Transgenic Broccoli Lines

Broccoli transformations were conducted according to the methodology provided by Sparrow and Irwin^14^ with minor modifications. The hypervirulent *A. tumefaciens* strain AGL1 was used for the transformation. Cotyledonary petioles from germinated seedlings of *B. oleracea* DH1012 were infected with AGL1 containing each of the binary vectors and then incubated on cocultivation medium at 26 °C for 3 days. Following cocultivation, cotyledons were transferred to Petri dishes containing the first-round selection medium supplemented with 15 mg/L kanamycin. After 3 weeks, the tissues were moved to fresh selection medium with 25 mg/L kanamycin. After an additional 3 weeks, green shoots were excised and transferred to a Phytatray II (Sigma) containing regeneration/rooting medium with 20–30 mg/L kanamycin. Once roots were established and leaves touched the Phytatray II lid, the plantlets were transferred to soil.

### Broccoli Genotyping

Broccoli lines were assessed for insertion of transgenes via PCR and gel electrophoresis using the Phire Plant Direct PCR Master Mix (Thermo Scientific). Leaf disks 2 mm in diameter were collected from broccoli leaves and placed in a dilution buffer before being heated at 100 °C for 2 min. Diluted plant DNA was then used in PCR reactions with primer sets capable of binding constructs A or C. PCR reactions were analyzed via gel electrophoresis using a 1% agarose gel in a Tris-acetate-EDTA (TAE) buffer. Stained agarose gels were imaged with a BioRad ChemiDoc instrument to determine the presence of transgene insertion.

### qPCR Analysis of Broccoli

Total mRNA was extracted using an E.Z.N.A. plant RNA kit (Omega Biotek) following manufacturer’s directions using the RB lysis buffer variation. Residual DNA was eliminated with a TURBO DNA-free kit (Thermo Fisher), and cDNA synthesis was achieved with an SSIV Vilo Master Mix kit using random hexamers (Thermo Fisher). Quantitative PCR was performed using a CFX96 Real-Time thermocycler (Bio-Rad) programmed for detection of SYBR intercalating dye with the following temperature programming: 95 °C for 3 min, then 95 °C for 30 s and 60 °C for 45 s repeated 39 times, and then a gradual increase from 65 to 95 °C at 0.5 °C/min to generate melt curves. Sso-Advanced Universal SYBR Green Supermix (Bio-Rad) was used for qPCR amplification. A previously validated primer set was used to amplify EF1α for internal normalization, and three sets of primers were tested for amplification of TGG4; the primer pair with the most consistent amplification was chosen. Melt curves for the product of EF1α (TGAGATGCACCACGAAGCTC and CCAACATTGTCACCAGGAAGTG) and TGG4 (GGTTCGCCCCGCTAAATGAATT and TGCTCAGGAGTGAATTCTGGCA) primer sets were unimodal and steep, suggesting that only a single product was formed. No reverse-transcriptase controls showed no amplification, confirming the efficacy of DNase treatment, and no template controls instituted at the beginning of RNA extraction with no plant matter and kept in parallel with real samples throughout all molecular steps did not amplify, confirming lack of contamination with extraneous DNA.

### Simulated Cooking and Mastication

Leaves of broccoli plants were harvested, placed in sealed plastic bags, and stored at 4 °C for no longer than 5 h before processing. Broccoli leaves were processed by removing the midrib and cutting the leaves into fourths. Each leaf section was randomly assigned a temperature treatment and placed in a labeled vacuum-sealed bag (Wevac). The bags were then vacuum-sealed using a vacuum sealer (ElecHomes). Vacuum bags containing broccoli leaves were then placed in a heated water bath (Thermo Scientific) at temperatures ranging from 30 to 100 °C for 10 min. Additionally, an uncooked control was included, which was not placed in a water bath but underwent all other procedures. Following heat treatment, vacuum-sealed broccoli leaves were flash-frozen in liquid nitrogen and stored at −80 °C until processed.

Frozen broccoli leaves were removed from their vacuum-sealed bags, placed in preweighed 2 mL screw cap tubes, frozen in liquid nitrogen, and lyophilized for 2 days (FreeZone 4.5, LabConco). Lyophilized broccoli leaves were then weighed to determine the dry mass of each tube. Lyophilized broccoli leaves were powderized using a ball mill (MM 400, Retsch) at 20 Hz for 10 min. To rehydrate the tissue for simulated mastication, 10 μL of water was added to each milligram of tissue. The mixture was homogenized by bead beating for 2 min at 30 Hz before being incubated at room temperature for 5 h. Following incubation, the broccoli samples were frozen in liquid nitrogen and lyophilized for 2 days.

### Metabolite Extractions and LC-MS Analysis

Extraction of metabolites from dried, masticated broccoli was conducted using 80% methanol and 20% water containing 5 ppm internal standard (12-[(cyclohexylcarbamoyl)amino]dodecanoic acid). For every 1 mg of broccoli tissue, 10 μL of an extraction solution was added. Samples were then homogenized via bead mill at 20 Hz for 10 min. Following homogenization, samples were centrifuged at 17000*g* for 10 min. The supernatant was then loaded into 96-well filter plates (0.22 μm, PVDF, Millipore) and centrifuged at 700*g* for 5 min.

LC-MS/MS analysis was performed as described by Barnum et al.^40^ with some modifications. Standard curves were generated using glucoraphanin, glucobrassicin, 4-methoxyglucobrassicin, gluconasturtiin, and sulforaphane for quantification. Data analysis was performed using MS-DIAL.^41^

### Plant Growth and Care

Transgenic broccoli plants were transplanted to 6 in. pots containing a soil composed of 50% peat and 50% sand and placed in a plant growth chamber. The chamber was set under a 16/8 h growth cycle. Daytime temperatures were 23 °C, and nighttime temperatures were 21 °C. Relative humidity was set to 60%. Plants were harvested at 4 months old after seed development had begun.

## Supporting Information Available

Data on additional enzymes tested, additional transgenic broccoli lines, and qPCR analysis of transgene expression(PDF)
