# Supplementary material for: Engineering Brassica Crops to Optimize Delivery of Bioactive Products Postcooking
Source: ACS Synth Biol. 2024 Feb 27;13(3):736–44. doi: 10.1021/acssynbio.3c00676 (PMC10949231; doi:10.1021/acssynbio.3c00676)
Supplement: Supplementary file 1 — sb3c00676_si_001.pdf [file sb3c00676_si_001.pdf]

Supplementary Materials for

Engineering *Brassica* crops to optimize delivery of bioactive products post-cooking

Authors: Collin R. Barnum, Myeong-Je Cho, Kasey Markel, and Patrick M. Shih

* Correspondence should be addressed to pmshih@berkeley.edu

- The supporting information contains data on additional enzymes tested, additional transgenic broccoli lines, and qPCR analysis of transgene expression.

This file includes:

Figures S1 to S6

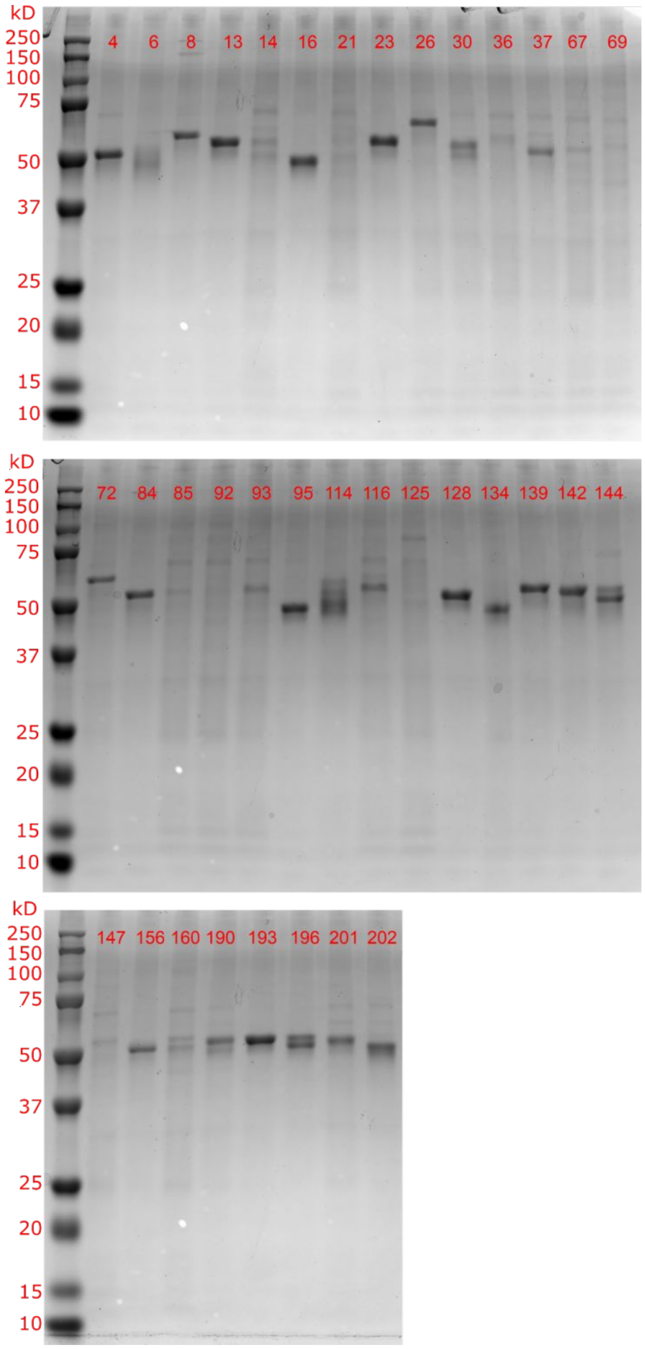

Figure S1. SDS-PAGE for all microbial family I glycosyl hydrolases expressed in *E. coli* and purified via IMAC.

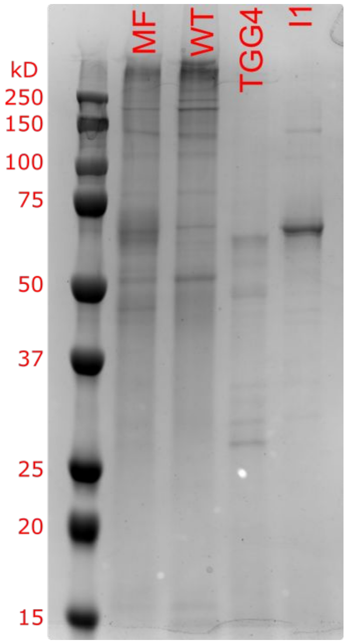

Figure S2. SDS-PAGE analysis of plant myrosinases following IMAC. Wildtype (WT) leaves of *N. benthamiana* were purified and analyzed alongside TGG4, I1 and MF461331 (MF).

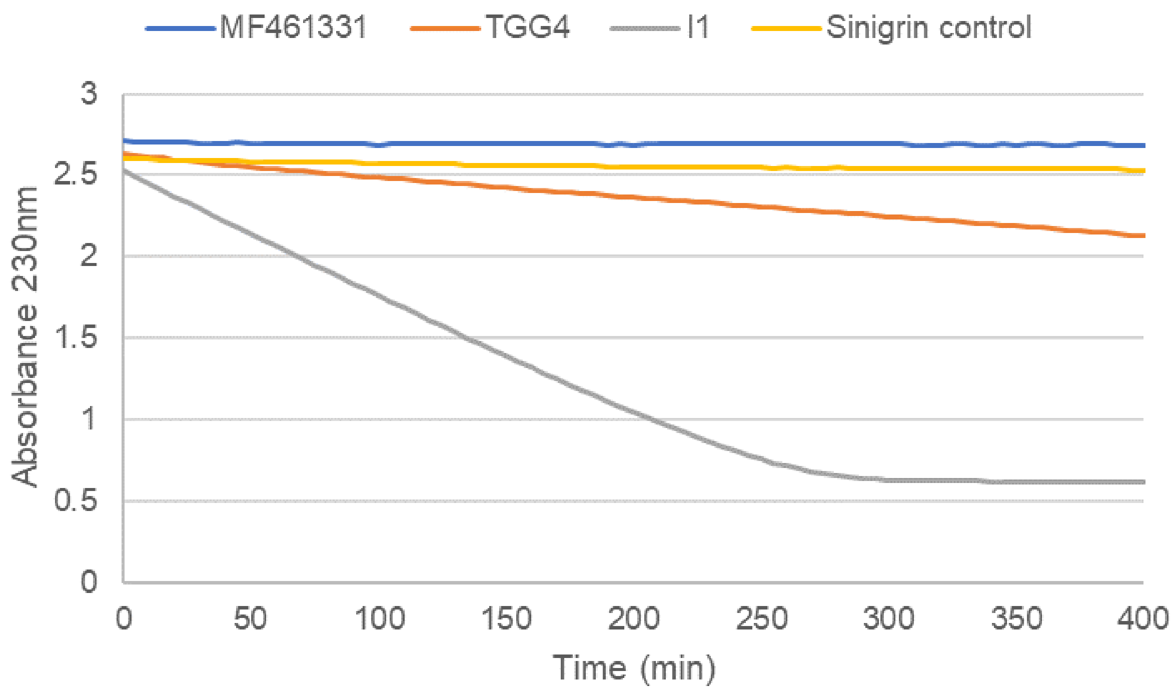

Figure S3. Determining activity of heterologously produced myrosinases. Absorbance at 230 nm was used to measure hydrolysis of sinigrin over time. Sinigrin control is a negative control with all components except a myrosinase.

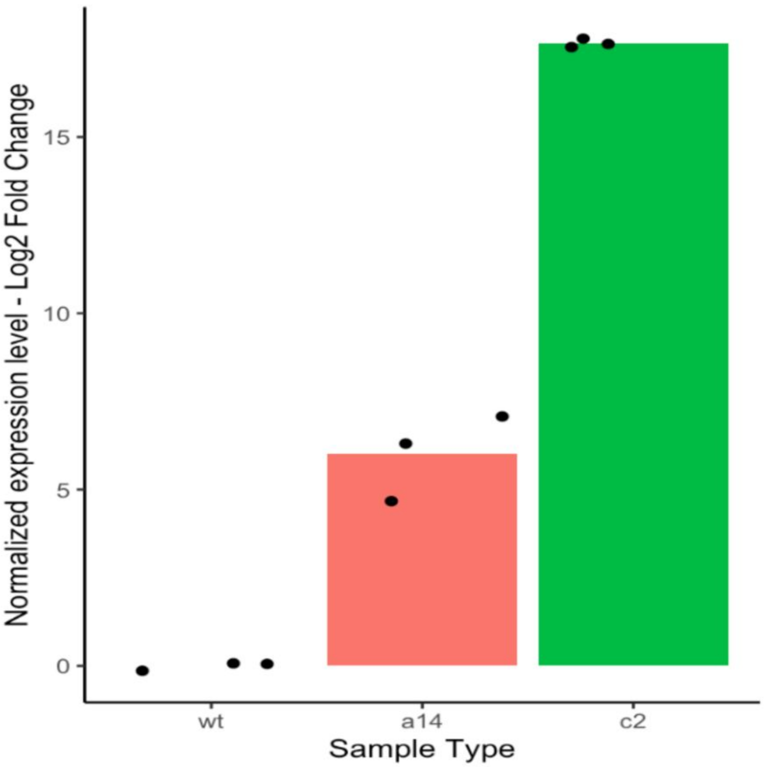

Figure S4. qPCR analysis TGG4 expression in transgenic broccoli. Normalized expression of TGG4 in two representative broccoli lines.

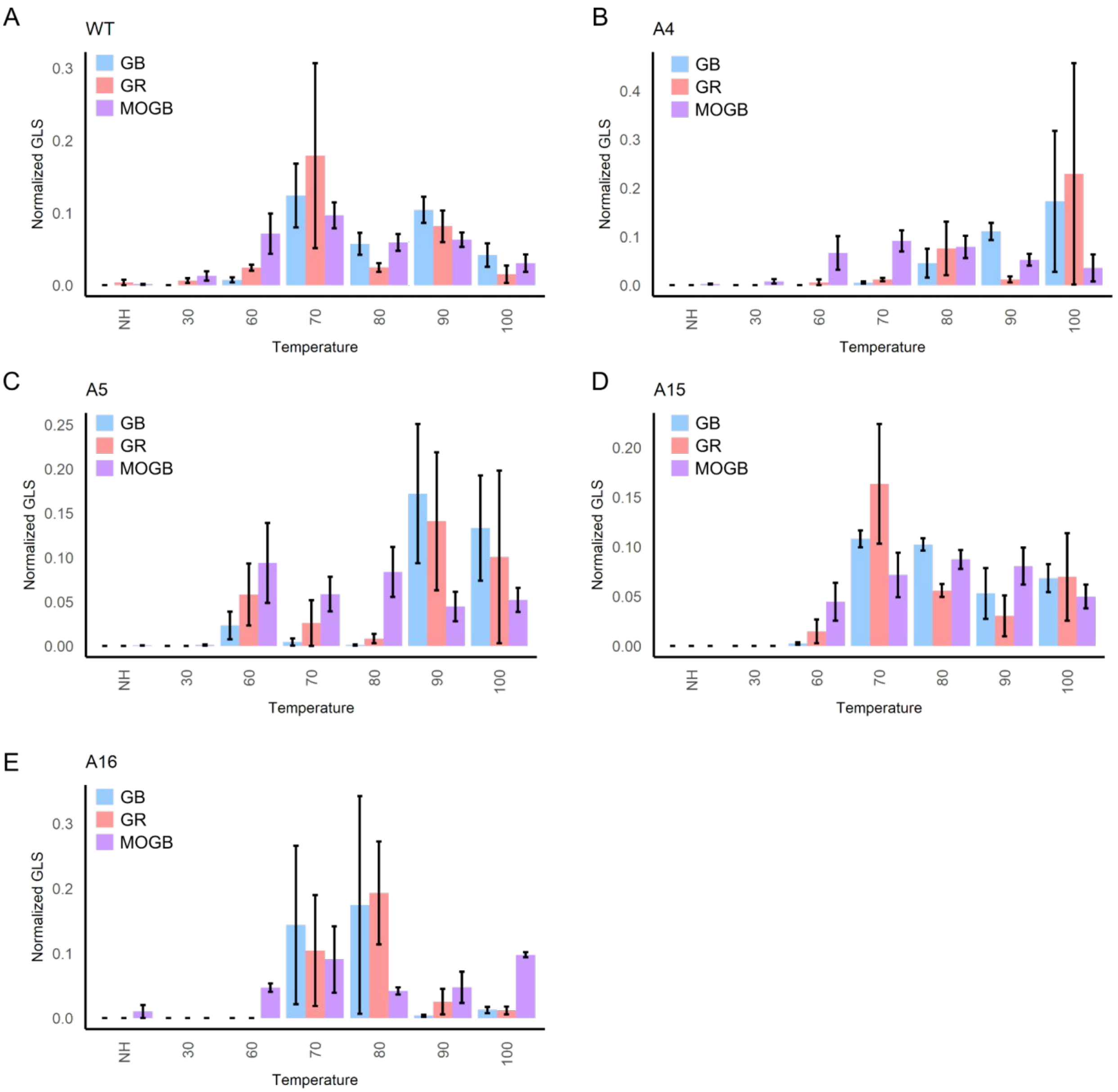

Figure S5. Normalized glucosinolate profile of select transgenic broccoli ines expressing TGG4 driven by pTGG1 after cooking at various temperatures. Plants from lines A) A4, B) A5, C) A15, D) A16, were analyzed via LC-MS. The glucosinolate content of each line was quantified and normalized to the total amount of glucosinolate present among all treatments to provide relative concentrations between treatments. All bars or dots represent the average normalized value of three leaf cuttings. Error bars represent the standard error of the mean.

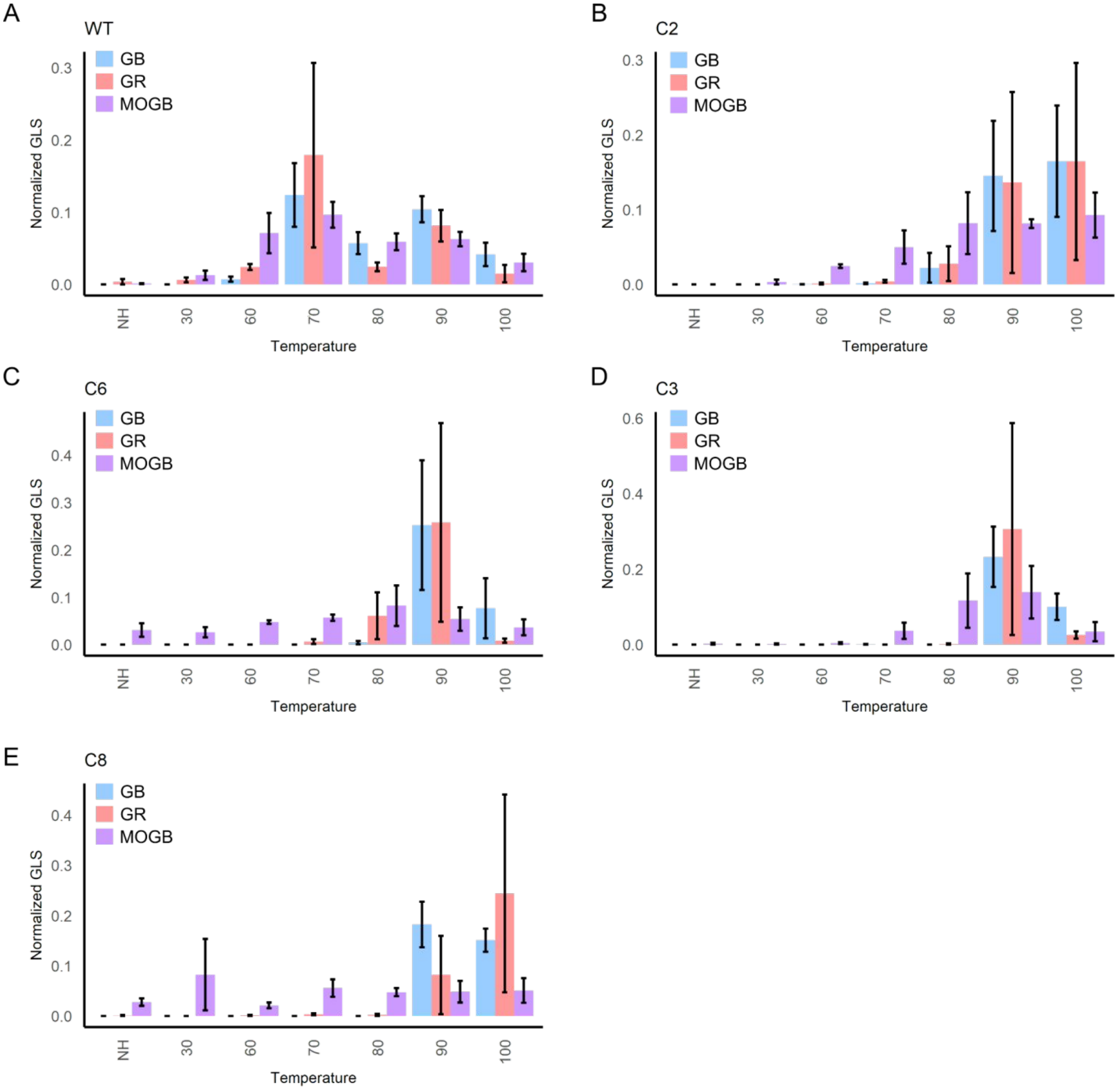

Figure S6. Normalized glucosinolate profile of select transgenic broccoli lines expressing TGG4 driven by p35S after cooking at various temperatures. Plants from lines A) C2, B) C6, C) C3, D) C8, were analyzed via LC-MS. The glucosinolate content of each line was quantified and normalized to the total amount of glucosinolate present among all treatments to provide relative concentrations between treatments. All bars or dots represent the average normalized value of three leaf cuttings. Error bars represent the standard error of the mean.
